# Illinois State Veterinary Medical Association

**Published:** 1893-01

**Authors:** Matthew Wilson

**Affiliations:** Recording Secretary


*Illinois State Veterinary Medical Association.* The tenth annual meeting of the Illinois State Veterinary Medical Association was held at the Sherman House, Chicago, November 16. The meeting was called to order by President S. S. Baker. The roll being called, the following members responded to their names: Drs. Walter Allen, A. G. Alverson, A. H. Baker, S. S. Baker, W. A. Baker, James Bond, G. Z. Barnes, H. E. Delavergne, T. J. Gunning, J. A. Judson, O. J. Lanigan, J. T. Nattress, C. A. Pierce, John Scott, J. S. Spangler, R. J. Withers, M. Wilson, R. G. Walker.

The minutes of the previous meeting were read and approved. The President then delivered his annual address.

It was moved by Dr. Barnes, seconded by Dr. Nattress, that the chair appoint a committee of three to draw up a report of sympathy and condolence on the death of Dr. C. S. Green, of Richmond, Illinois, and report at the next session. Motion carried. The Chair appointed as such committee Drs. Alverson, Barnes and Nattress.

The Treasurer's report showed a balance on hand of $40.32.

Report of committee on fee bill was deferred till next session.

The President then called on Dr. Allen for his paper on "A Few Things I Want to Know." Discussion was closed on motion.

J. H. Bates, M.D., of Neponsit, being absent, but having sent his paper on "Shall we know each other better?" the Secretary was called upon to read it.

Dr. Wilson taking the chair, Dr. Baker read his paper on "Antiseptic Surgery." Discussion was closed on motion

The following gentlemen's names were proposed for membership: Drs. J. W. Kaul, Chicago; J. L. Siegresser, Chicago; G. W. Pope, Chicago; J. G. Fish, Chicago; G. S. Baker, Chicago; Charles Spielman, Chicago; G. W. Browning, Lincoln.

On motion of Dr. Scott, seconded by Dr. Walker, the rules were suspended for the time being, and the above-named gentlemen elected by acclamation.

Bills to the amount of $22.40 were audited and ordered paid.

The motion to adjourn until next morning at 10 o'clock was carried.

NOV. 17. The meeting was called to order by the President, Dr. Baker.

Committee on fee bill reported through its chairman, Dr. Nattress. On motion of Dr. Wilson, seconded by Dr. Alverson, the report of the committee was adopted and the committee discharged.

The Committee on Legislation reported through its chairman, Dr. Walker. On motion of Dr. Scott, seconded by Dr. Gunning, the report of the committee was adopted and the committee discharged.

The Committee on Membership, through its chairman, Dr. Alverson, reported a number of new members admitted and new applicants for membership.

On motion of Dr. Scott, seconded by Dr. Gunning, the report of the committee was adopted and the committee discharged.

Dr. Hollingsworth being absent, the Secretary read his paper on the report of two rare cases.

The President then called on Dr. Nattress for his paper on "Azoturia." The usual lengthy discussion on this disease followed, which was closed on motion.

Dr. Walker then read his paper on "Veterinary Legislation," and was followed by J. A. McDonell, M.D., on "Foot and Mouth Disease in Men and Animals."

Dr. W. L. Williams, of Purdue University, Lafayette, Indiana, President of the United States Veterinary Medical Association, was then called upon and spoke regarding the International Congress of Veterinary Surgeons to be held in Chicago in 1893, and was followed by Professors A. H Baker and R. J. Withers, of the Chicago Veterinary College, on the same subject.

The following gentlemen's names were proposed for membership: Drs. V. E. Frizell, La Moille; J. Henderson, Chicago; J. Fabian Magor, Chicago; W. S. Brayton, Blue Island.

On motion of Dr. Walker, seconded by Dr. Scott, the rules were suspended for the time being and the above-named gentlemen elected by acclamation.

The annual election of officers resulted in the following being elected:

President, Dr. S. S. Baker; Vice President, Dr. John Scott; Secretary, Dr. M. Wilson; Treasurer, A. G. Alverson.

*Board of Censors:* Drs. Walker, Gunning and Brayton.

The following standing committees were appointed:

*Committee on Program:* Drs. S. S. Baker *ex officio*, Wilson, Stringer, Nattress and Dietwig.

*Committee on Arrangements:* Drs. John Scott *ex officio*, Frizell and Story.

*Committee on Legislation:* Drs S. S. Baker, Walker, Lanigan, Nattress, Alverson, Wilson, Spielman and Ryan.

*Committee on Membership:* Drs. Bond, Pierce, Hollingsworth, Kaull and Whitmore.

*Committee on International Entertainment:* Drs. S. S. Baker, John Scott, Walker, Gunning, Wilson, Alverson, Withers, A. H. Bakers, J. F. Ryan, Hughes, Allen, Hollingsworth, Nattress, Barnes. Melvin and G. W. Pope.

Moved by Dr. Walker, seconded by Dr. Scott, that the committee have full power to act. Carried.

It was moved by Dr. Scott, seconded by Dr. Walker, that a special assessment of $5.00 per member be levied for the entertaining of the International Congress. Carried.

It was moved by Dr. Spielman, seconded by Dr. Nattress, that a special assessment of $5.00 per member be levied for the purpose of legislation. Carried.

It was moved by Dr. Walker, seconded by Dr. Scott, that we have two hundred and fifty copies of the Constitution and By-Laws printed for distribution among the members. Carried.

It was moved by Dr. Gunning, seconded by Dr. Allen, that a form of Certificate of Membership of the Association be drafted and presented at the next meeting. Carried.

The Committee on Resolutions of Sympathy and Condolence on the death of

C. S. Green then reported through its chairman, Dr. Alverson. The report was adopted and the committee discharged.

The meeting adjourned to meet in Peoria, Illinois, next February at the call of the committee.

MATTHEW WILSON, M.R.C.V.S., *Recording Secretary.*

